# Mandibular Cemento‐Osseous Dysplasia: A Report of Three Cases in Indian Women With Emphasis on Diagnostic Challenges

**DOI:** 10.1155/crid/7118123

**Published:** 2026-10-01

**Authors:** Suganya Ramalingam, Sharada T. Rajan, Vandana Sampath, Sharon Sam

**Affiliations:** ^1^ Department of Oral Pathology Sri Ramachandra Dental College and Hospital Sri Ramachandra Institute of Higher Education and Research Chennai India sriramachandra.edu.in

**Keywords:** fibro-osseous lesions, florid cemento-osseous dysplasia, gender, health care, periapical cemento-osseous dysplasia, public health, well-being, women

## Abstract

Cemento‐osseous dysplasia (COD) is a benign fibro‐osseous lesion of the jaw bones that usually affects females and is often seen incidentally on radiographic examination. Its varied clinical and radiographic presentation resembles odontogenic cysts and other fibro‐osseous lesions, making diagnosis challenging. This paper highlights three cases of cemento‐osseous dysplasia, each with distinct clinical manifestations. These cases demonstrate the varied presentations of cemento‐osseous dysplasia, underscoring the need for combined clinical, radiological, and histopathological evaluation for accurate diagnosis.

## 1. Introduction

Cemento‐osseous dysplasias (CODs) are a group of benign fibro‐osseous lesions of the jaws categorized by the replacement of normal bone with a combination of fibrous connective tissue and mineralized material mimicking bone and/or cementum. They are nonneoplastic, localized to tooth‐bearing areas, and are thought to originate from the periodontal ligament [1].

COD tops the list of six main fibro‐osseous lesions in the 2022 WHO head and neck tumor classification. Despite being a nonneoplastic, jaw‐specific lesion, recent research reveals COD involves pathogenic mutations in the RAS‐MAPK pathway, including BRAF, HRAS, KRAS, NRAS, and FGFR3 [2]. Even though COD represents the most common fibro‐osseous lesion of the jaws, its prevalence in the general population remains relatively low, ranging from approximately 0.2% to 5.5%, with a strong predilection for middle‐aged women of African descent [3].

It is categorized into three subtypes—periapical, focal, and florid COD—based on the location and extent of the lesion. CODs show a marked predilection for the mandible and are frequently identified as incidental findings on radiographic examination owing to their typically asymptomatic behavior [4]. When symptomatic, presentation ranges from an absence of symptoms to localized pain or tenderness associated with secondary changes such as infection [5].

Various reports from Asian and Indian populations remain limited, particularly for florid COD, which is considered the least frequent subtype. This relative infrequency, coupled with its variable radiographic appearance, contributes to frequent diagnostic confusion with odontogenic cysts and other fibro‐osseous lesions. Radiographically, these lesions show a variety of appearances, ranging from radiolucent to mixed and radiopaque stages, reflecting their progressive maturation [3]. Histopathologically, these lesions reveal a cellular fibrous stroma showing woven bone, lamellar bone, and cementum‐like calcifications in variable proportions. Because CODs may mimic various other fibro‐osseous lesions such as cemento‐ossifying fibroma, and also odontogenic cysts and tumors, residual cysts, and aneurysmal bone cysts, clinico‐radiological and histopathological correlation is vital for precise diagnosis and appropriate management [6]. The present case series highlights the varying clinical presentations and diagnostic challenges associated with different forms of COD. This case series is aimed at comparing the clinical, radiographic, and histopathological features of the focal, periapical, and florid COD subtypes encountered in this cohort, in order to outline diagnostic points that may help clinicians differentiate COD from radiographically similar jaw lesions.

## 2. Case Series

### 2.1. Case 1

A 57‐year‐old female patient, with no relevant habits or past medical history, presented with the chief complaint of missing lower teeth. Clinical examination revealed no abnormalities in the oral cavity, and the surrounding gingiva showed no signs of ulceration or inflammation.

Radiographic evaluation, including CBCT advised for implant planning, revealed a well‐defined mixed radiolucent–radiopaque lesion in the region of Tooth 43 (Figure 1A, CBCT sectional image). Based on these findings, the surgeon considered a residual (remnant) cyst as the initial differential diagnosis and decided to surgically excise the lesion.

**Figure 1:**
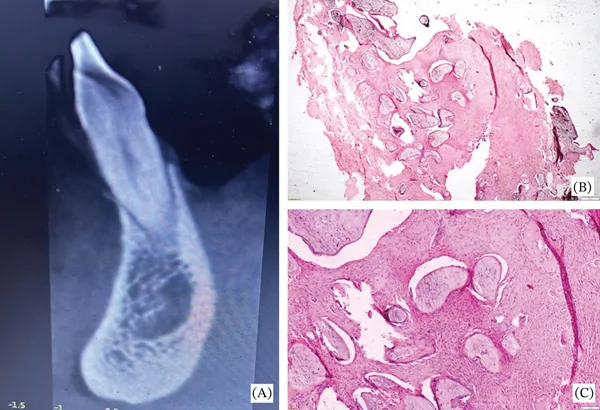
Case 1 (focal cemento‐osseous dysplasia). (A) Parasagittal CBCT sectional image corresponding to the region of Tooth 43, showing a well‐defined mixed radiolucent–radiopaque lesion with intact cortical boundaries and altered internal trabecular architecture; the crown and root of Tooth 43 are not directly visualized within this section plane. (B,C) Photomicrographs showing a connective tissue stroma with plump fibroblasts, bony trabeculae containing osteocytes, and basophilic cementum‐like calcifications (H&E stain; 10x and 40x view).

The excised tissue was fixed in formalin and submitted for histopathological examination. Microscopic analysis showed a connective tissue stroma composed of plump fibroblasts, along with trabeculae of bone containing osteocytes and areas of basophilic calcifications resembling cementum (Figure 1B,C). By correlating the radiographic and histopathological features, a final diagnosis of focal COD was made.

### 2.2. Case 2

A 39‐year‐old female patient reported with a chief complaint of sensitivity in lower teeth region for the past 1 year. The sensitivity was gradual in onset and persistent in nature. There was no history of acute pain, pus discharge, or trauma associated with the affected region. (Figure 2A,B). The patient did not report any noticeable swelling or rapid increase in size of the jaw during this period.

**Figure 2:**
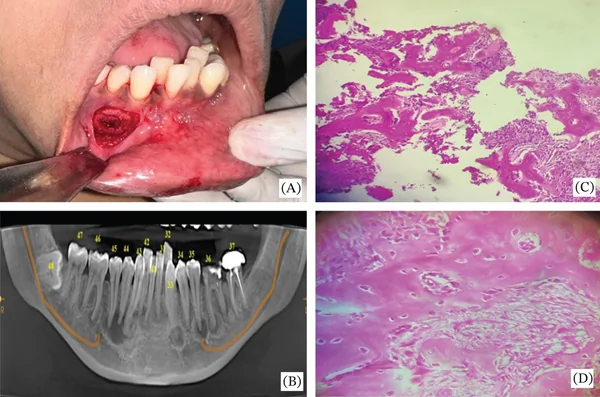
Case 2 (periapical cemento‐osseous dysplasia). (A) Intraoral clinical photograph of the mandibular anterior region. (B) CBCT panoramic reconstruction demonstrating bilateral, multifocal, mixed radiolucent–radiopaque periapical lesions involving the mandibular anterior teeth; Tooth 41 is indicated at the midline. (C,D) Photomicrographs showing islands of woven bone with focal cementum‐like calcifications in a richly cellular fibrous stroma with extensive hemorrhage (H&E stain; 20x view).

Clinical examination of the lower jaw revealed mild tenderness in the involved area, with no obvious facial asymmetry. Intraorally, the overlying mucosa appeared normal, and the teeth in the affected region were present and vital. Radiographic evaluation, including CBCT panoramic reconstruction, revealed bilateral, multifocal, mixed radiolucent–radiopaque lesions involving the periapical region of the mandibular anterior teeth (Figure 2B). Based on these clinical and radiographic findings, a provisional diagnosis of periapical inflammatory lesions versus early‐stage periapical COD was considered.

Histopathological examination revealed islands of woven bone with focal areas of cementum‐like calcifications within a connective tissue stroma. The stroma was richly cellular, composed predominantly of spindle‐shaped fibroblasts, and was surrounded by extensive areas of hemorrhage, supporting the diagnosis of a periapical COD. (Figure 2C,D).

### 2.3. Case 3

A 28‐year‐old female patient presented with a chief complaint of dull, persistent pain in the anterior region of the mandible for the past 1 year (Figure 3A). The pain was gradual in onset, nonradiating, and not associated with any episodes of acute exacerbation. There was no history of trauma, pus discharge, or parafunctional habits. The patient′s medical history was noncontributory.

**Figure 3:**
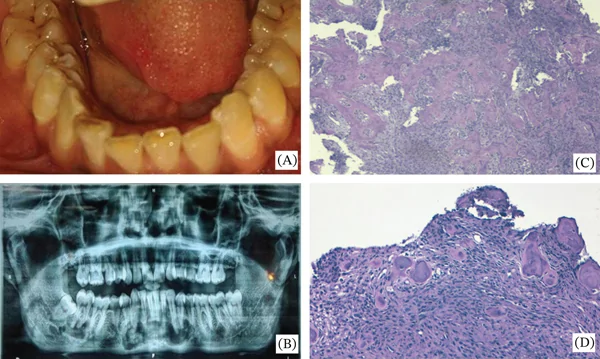
Case 3 (florid cemento‐osseous dysplasia). (A) Intraoral clinical photograph of the mandibular anterior region. (B) Panoramic radiograph showing multiple mixed‐density lesions scattered throughout the mandible, consistent with florid cemento‐osseous dysplasia; a separate periapical/pericoronal radiolucency with localized cortical expansion is also noted distal to the impacted mandibular left third molar. (C,D) Photomicrographs showing a fibrocellular stroma with irregular trabeculae of woven and lamellar bone and basophilic cementum‐like calcifications, without a capsule or neoplastic growth pattern (H&E stain; 10x and 40x view).

On clinical examination, no facial asymmetry or extraoral swelling was evident. Intraoral examination revealed intact overlying mucosa with no signs of ulceration or inflammation. The teeth in the affected region were present and responded positively to vitality testing, and no abnormal tooth mobility was noted.

Radiographic evaluation revealed a well‐defined mixed radiolucent–radiopaque lesion involving the lower anterior region, with expansion of the involved bone (Figure 3B). A separate, well‐defined periapical/pericoronal radiolucency with localized cortical expansion was also noted distal to the impacted mandibular left third molar; this finding was considered unrelated to the anterior lesion and to the subsequent diagnosis of florid COD and was managed by clinical and radiographic follow‐up rather than active intervention. Considering the presence of the anterior mixed‐density lesion with perceived expansile characteristics, a provisional diagnosis of cemento‐ossifying fibroma was given.

However, histopathological examination demonstrated a fibrocellular connective tissue stroma containing irregular trabeculae of woven and lamellar bone along with multiple basophilic cementum‐like calcifications, without evidence of a well‐formed capsule or neoplastic growth pattern (Figure 3C,D). These features were inconsistent with cemento‐ossifying fibroma.

Further correlation with radiological findings, including the distribution pattern and absence of significant cortical expansion, supported a diagnosis of florid COD. The multifocal nature and maturation pattern of the lesion favored florid COD over a true neoplastic fibro‐osseous lesion.

## 3. Discussion

This article describes the case series of Indian women affected by various types of COD, representing a characteristic clinical depiction for the general dental practitioner. The clinical, radiographical, and histopathological diagnosis, along with management and follow‐up have been discussed in detail.

In Case 1, a mixed radiolucent–opaque lesion linked to missing mandibular teeth was primarily assumed to be a residual cyst. Incisional biopsy was done to rule out cystic pathology. Histopathological examination showed the fibroblastic stroma with bone and cementum‐like calcifications, suggesting the confirmatory diagnosis of focal COD. This highlights how important it is to pair the radiological interpretation with tissue histopathology to differentiate COD from other lesions [7]. However, surgical complication such as osteomyelitis may include, whereas the patient in the present case, no complications were observed a few months after the biopsy.

Case 2 presented with prolonged sensitivity and bilateral, multifocal periapical lesions initially considered to represent periapical inflammatory lesions versus early‐stage periapical COD. However, histopathology revealed woven bone with cementum‐like areas within a cellular fibrous stroma, confirming a diagnosis of periapical COD. Periapical COD often mimics periapical inflammatory lesions radiographically, particularly in early radiolucent stages, underscoring the role of vitality testing and histopathologic correlation for accurate diagnosis [5, 8].

In Case 3, symptoms and radiographic features suggested cemento‐ossifying fibroma; however, further correlation revealed patterns consistent with florid COD, the subtype characterized by multifocal involvement. Florid COD can mimic other fibro‐osseous lesions and odontogenic tumors both radiographically and clinically [5, 6].

Recent radiologic studies show that mandibular buccal cortical plate is the most commonly affected bone, with irregular/circular shaped lesions and well‐defined margins. Based on the volume of the lesion, damage to the cortical bone is determined and discontinuity of the lamina dura of involved teeth is noted [9].

Florid COD was first formally delineated by Melrose et al. in 1976; earlier reports of comparable jaw lesions were largely subsumed under the older term “cementoma” [10]. Comparative data further indicate a lower reported prevalence of COD among Asian and Indian populations relative to cohorts of African descent, which may partly reflect underrecognition and lower rates of incidental CBCT imaging in resource‐limited settings [3, 9].

Comparing the three cases directly, each subtype followed a distinct diagnostic trajectory: The focal lesion in Case 1 and the periapical lesions in Case 2 were each initially mistaken for cystic pathology because of their radiolucent or mixed radiographic appearance, whereas the florid lesion in Case 3 was initially mistaken for a neoplastic fibro‐osseous lesion because of its expansile, mixed‐density appearance. In all three cases, the correct diagnosis was reached only after histopathological correlation, underscoring that radiographic appearance alone, irrespective of COD subtype, is insufficient for diagnosis. This comparison directly supports the aim of the present series: A structured approach combining clinical history, multiplanar radiographic assessment (preferably CBCT), and histopathological correlation should be applied uniformly across COD subtypes to avoid both misdiagnosis and unnecessary surgical intervention.

Accurate diagnosis requires combining clinical, radiographic, and histopathological findings. Recognizing COD is crucial to avoid misdiagnosis and overtreatment, as most cases need conservative management and follow‐up, not an aggressive surgical procedure. This series stresses the need for a multidisciplinary approach to diagnose and manage COD effectively. Complications may include secondary infection or osteomyelitis mainly in florid COD.

Precise diagnosis of COD is important, as these lesions are characteristically self‐limiting and frequently require observation rather than aggressive surgical procedures. Biopsy is warranted when radiographic and clinical features are unclear, but unnecessary treatment should be avoided to lessen complications such as delayed healing.

## 4. Conclusion

We presented a series of cases highlighting the diagnostic challenges of COD variants (focal, periapical, and florid) in women across various ages. A combination of clinical, radiographical, and histopathological findings needs to be performed to arrive at the correct diagnosis. Understanding the features of COD can guarantee management and follow‐up while preventing needless surgical intervention.

## Funding

No funding was received for this manuscript.

## Consent

Written informed consent was obtained from all three patients.

## Conflicts of Interest

The authors declare no conflicts of interest.

## Supporting information


**Supporting Information** Additional supporting information can be found online in the Supporting Information section. Document S1: CARE checklist of information to include when writing a case report.

## Data Availability

Data sharing is not applicable to this article as no datasets were generated or analyzed during the current study.
